# Risk factors for hearing loss in children: a systematic literature review and meta-analysis protocol

**DOI:** 10.1186/s13643-019-1073-x

**Published:** 2019-07-17

**Authors:** Bénédicte Vos, Dorie Noll, Marie Pigeon, Marlene Bagatto, Elizabeth M. Fitzpatrick

**Affiliations:** 1 Faculty of Health Sciences, University of Ottawa, 451 Smyth Road, Ottawa, Ontario K1H 8M5 Canada; 2 Child Hearing Laboratory, CHEO Research Institute, 401 Smyth Road, Ottawa, Ontario K1H 8L1 Canada; 3 School of Public Health, Université libre de Bruxelles (ULB), Route de Lennik 808 CP 598, 1070 Brussels, Belgium; 4 Audiology Department, CHEO, 401 Smyth Road, Ottawa, Ontario K1H 8L1 Canada; 5 School of Communication Sciences and Disorders and the National Centre for Audiology, Western University, 1201 Western Road, London, Ontario N6G 1H1 Canada

**Keywords:** Hearing loss, Congenital hearing loss, Late onset hearing loss, Acquired hearing loss, Progressive hearing loss, Risk factor, Surveillance, Newborn, Children

## Abstract

**Background:**

Hearing loss in newborns and children is a public health concern, due to high prevalence and negative effects on their development. Early detection and intervention of childhood hearing loss may mitigate these negative effects. Population-based newborn hearing screening programs have been established worldwide to identify children at risk for congenital hearing loss and to follow children at risk for late onset or progressive hearing loss. This article presents the protocol for a systematic review that aims to review the risk factors associated with permanent hearing loss in children, including congenital, early, or late onset. Risk factors associated with progressive hearing loss will be investigated as a secondary aim.

**Methods:**

Scientific literature from the following databases will be investigated: MEDLINE, Ovid MEDLINE(R) Daily and Ovid MEDLINE(R), Embase, and CINAHL. The primary outcome is a permanent bilateral or unilateral hearing loss with congenital onset or onset during childhood (birth to 18 years). The secondary outcome is progressive hearing loss. Studies must report data on risk factors associated with permanent hearing loss; risk factors may be present at birth or later and result in immediate or delayed hearing loss. Randomized controlled trials, quasi-experimental studies, nonrandomized comparative and non-comparative studies, and case series will be included. The risk of bias will be assessed using the Qualitative Assessment Tool for Quantitative Studies (McMaster University). If aggregation of data is possible for a subsection of studies, we will pool data using meta-analysis techniques. If aggregation of data is not possible, a qualitative synthesis will be presented. We will assess the quality and strength of the overall body of evidence using the Grading of Recommendations Assessment, Development and Evaluation (GRADE). The systematic review follows the Preferred Reporting Items for Systematic Reviews and Meta-Analyses (PRISMA) recommendations.

**Discussion:**

The resulting information will inform the update of a provincial audiological surveillance protocol for the Ontario Infant Hearing Program and will be applicable to early hearing detection and intervention (EHDI) programs worldwide.

**Systematic review registration:**

We have registered the protocol in the International Prospective Register of Systematic Reviews (PROSPERO), registration number CRD42018104121.

**Electronic supplementary material:**

The online version of this article (10.1186/s13643-019-1073-x) contains supplementary material, which is available to authorized users.

## Background

Permanent hearing loss affects at least 1 to 2 per 1000 children at birth and has severe consequences for their development [1–3]. The negative consequences for language, cognitive, and social-emotional skills are particularly important when diagnosis is delayed for children with hearing loss, which delays access to early intervention programs [4–7]. There is good evidence that universal newborn hearing screening (UNHS) programs achieve early identification [8, 9]. When UNHS programs are implemented as part of a comprehensive early hearing detection and intervention program (EHDI), children with congenital and early onset hearing loss are diagnosed early and supported to develop language in their first months of life [3, 10].

In addition to screening newborns for hearing loss, there is a need to monitor children who are at risk of developing hearing loss after the neonatal period [11–13]. Prior to the population-based screening, it was difficult to identify whether hearing loss occurred congenitally, neonatally, or during early childhood beyond the neonatal period. Studies suggest that the prevalence of permanent hearing loss rises during childhood and that hearing loss occurs sometime after infancy in up to 25–50% of affected children [14–16]. Almost half of the children with hearing loss will experience deterioration in hearing during childhood [17, 18].

Children at risk for late onset hearing loss need to be closely monitored, even after passing a screening test, to ensure identification as early as possible and to avoid delays in treatment, unnecessary costs to the healthcare system, and additional parental stress [19, 20]. It is also important to monitor children with early onset hearing loss who are at risk for further deterioration in hearing (progressive hearing loss), so that appropriate intervention may be initiated in a timely manner. Surveillance programs are thus recommended as part of a comprehensive EHDI program but not universally applied [21, 22]. They aim to identify children at risk for late onset or progressive hearing loss and have been implemented in some UNHS or EHDI programs [11, 23, 24]. However, the effectiveness of these programs is not well established [25].

In 2007, the Joint Committee on Infant Hearing (JCIH) updated the list of risk factors associated with permanent congenital, delayed onset, or progressive hearing loss in childhood [21]. Due to the overlap between congenital/neonatal and delayed onset risk factors, the JCIH presented a single list. The JCIH recommends that all infants with these risk factors be closely monitored for hearing loss. The committee proposed a comprehensive diagnostic assessment in the neonatal period following a positive screening result and appropriate surveillance after a negative screening result. The JCIH list is widely adopted in a large number of UNHS programs, sometimes with adaptations. However, this list was published more than 10 years ago. Based on the evolution of care and new scientific knowledge during recent years, updating the risk factors to reflect current clinical practice is warranted [16]. Our systematic review aims to identify the most up-to-date risk factors for hearing loss in children and will include an examination of factors by the onset of hearing loss.

## Methods

### Objectives and research questions

The overall aim of this systematic review is to synthesize evidence on risk factors related to neonatal, early, and late onset hearing loss as well as progressive hearing loss in children. We will investigate all risk factors that have an immediate or delayed effect on hearing. The resulting information will inform surveillance protocols and will be applicable to EHDI programs worldwide.

The review aims to answer the following primary research question: what risk factors are associated with permanent hearing loss in children? Risk factors related to congenital, early, and late onset hearing loss will be examined.

A secondary objective of this review is to investigate the risk factors associated with progressive hearing loss in children with congenital, early, or late onset hearing loss.

### Design

This systematic review protocol has been developed according to the Preferred Reporting Items for Systematic review and Meta-Analysis Protocols (PRISMA-P) (see Additional file 1). We have registered the protocol in the International Prospective Register of Systematic Reviews (PROSPERO), registration number CRD42018104121) [26].

### Eligibility criteria

Primary research that meets the following criteria will be included in the systematic review.

#### Study designs

It is anticipated that literature in this field will consist largely of observational and descriptive studies. However, some risk factors are related to medical conditions or treatments and may be investigated through intervention studies. In an effort to collect the most comprehensive data, the following study designs will be included: randomized controlled trials, quasi-experimental studies, nonrandomized comparative studies (prospective, or retrospective cohort, case-control), nonrandomized studies without comparison group (e.g., prospective or retrospective cohort, cross-sectional), and case series. Consequently, we will exclude from this review case reports/case studies (less than five cases). Since we anticipate a very large number of articles, we have essentially extended the “case study” to include < 5 cases as we judged that these small cohorts would provide little additional information. We will also exclude editorials, viewpoints/opinion, literature reviews, conference abstracts, guidelines, recommendations, consensus papers, qualitative research, and gray literature, such as reports, thesis, and notes.

Studies not directly related to the review questions but that present pertinent information about the relationships between risk factors and hearing loss will also be included, as long as they meet the review inclusion criteria (e.g., population, exposure, outcomes).

#### Population

All children with permanent bilateral or unilateral hearing loss will be included. For this review, a child is defined as age birth to 18 years. In most healthcare systems, pediatric care targets children from birth to adolescence (defined as less than 19 years), which is concordant with the Medical Subject Heading (MeSH) definition (https://www.ncbi.nlm.nih.gov/mesh/). The age limit is because only the childhood onset of hearing loss is targeted by the research question. We will include articles on children and adults only if results are presented separately for children; we will accept an upper range of the study group of approximately age 21 years (young adults), when the majority of participants are children.

#### Exposure

Studies must report data on risk factors associated with permanent hearing loss. Examples of specific risk factors are congenital cytomegalovirus (cCMV), toxoplasmosis, admission to a neonatal intensive care unit, ototoxic medications, and a family history of hearing loss [21]. Risk factors may be present at birth (e.g, cCMV, family history) or can present later in infancy or childhood (e.g., hyperbilirubinemia, meningitis). Hearing loss associated with these risk factors may have congenital onset, late onset, or be progressive in nature.

Risk factors associated with temporary conductive hearing loss will be excluded. Articles reporting “infants at risk” will not be eligible; the specific risk factor(s) must be identified.

#### Comparators

For studies that include a comparison group, the comparator will be either (1) no risk factors or (2) a comparison between one risk factor and other factors. Due to the nature of studies in this field, it is anticipated that most studies will not include a comparison group.

#### Outcome

##### Primary outcome

The primary outcome is permanent bilateral or unilateral hearing loss in childhood. Permanent hearing loss refers to sensorineural hearing loss or conductive loss that is structural in nature and expected to last for 6 months or more [24]. Therefore, studies reporting outcomes as sensorineural hearing loss, auditory neuropathy spectrum disorder, permanent conductive hearing loss or mixed loss will be included. Definitions of hearing loss (i.e., the severity of hearing loss) will be accepted as defined by authors. Our interest in this review is only on peripheral hearing loss; therefore, studies reporting auditory processing disorders (or central auditory processing) will be excluded.

We will categorize the outcomes according to the onset of hearing loss:

i) Congenital and early onset is defined as hearing loss present at birth or diagnosed within the first 3 months of life. This definition is consistent with the JCIH (2007) benchmark of diagnosis by 3 months of age [21].
ii) Late onset is defined as the occurrence of hearing loss, typically after 3 months of age, after a normal hearing screen or confirmation of normal hearing through audiologic assessment.

The flexibility in these definitions allows for known variations in the classification of onset of hearing loss in the literature. Studies that report exclusively on temporary conductive hearing loss or change in hearing following surgical procedures (e.g., middle ear surgery, cochlear implants) are not targeted by this review.

The diagnosis of hearing loss must be confirmed by appropriate assessment methods at the time of the study. Results from automated tests used for hearing screening (such as automated otoacoustic emissions or automated auditory brainstem responses) will be excluded. Outcomes based solely on parental report of hearing loss will also be excluded. Hearing loss defined as “requiring hearing aids/amplification” will be accepted as a permanent hearing loss only if assessed by audiologists or specialized professionals, based on appropriate tests.

##### Secondary outcome

The secondary outcome of interest for this review is progressive hearing loss. Any amount of progressive hearing loss as defined by the authors will be included. All other inclusion criteria detailed under the primary outcome will apply.

### Search method and information sources

The search strategy was developed in collaboration with a health sciences librarian experienced in systematic reviews. The following databases will be searched: MEDLINE including Epub Ahead of Print, In-Process & Other Non-Indexed Citations, Ovid MEDLINE(R) Daily and Ovid MEDLINE(R) (1946 to July 30 2018), and Embase (1946 to July 30 2018) using the Ovid interface. CINAHL will be searched from inception on July 23, 2018 (database coverage dates not stated). Searches will be limited to the cohort, case control, or randomized controlled trials using existing filters. We will consider exposure of all risk factors outlined in the provincial surveillance protocol for the Ontario IHP [24]. We will also include general terms such as surveillance, risk factor, or risk indicator to capture risk factors that may not be included on the Ontario IHP list. The search strategy is presented in Additional file 2.

Restrictions will apply to language and publication date. We will consider papers published only in English since 1990, that include birth cohorts ≥ 1985. Articles in other languages will be screened only at the broad screening level. The date limit was set to correspond broadly with the implementation of UNHS programs.

### Selection process and data management

The study selection involves three stages, managed within covidence (covidence systematic review software, http://www.covidence.org). Prior to the title and abstract screening, a screening form will be developed based on the inclusion and exclusion criteria and will be tested among reviewers with a subset of articles. Titles and abstracts will be assessed by two independent reviewers (BV and EF or DN) for potential relevance; the reviewers will resolve any conflicts (e.g., one reviewer selected “yes” or “maybe” and the other selected “no”) and involve a third reviewer if required. At the second stage, two independent reviewers (BV and EF or DN) will screen all potentially relevant full-text articles (all articles tagged as “yes” or “maybe” during the previous stage). Disagreements will be resolved by consensus or a third member of the research team.

All processes will ensure that bias is minimized when deciding whether to include or exclude certain studies, based on the application of objective inclusion/exclusion criteria, independent reviewers, and conflicts resolved through a third reviewer.

A PRISMA flow chart will present the number of studies included/excluded in each stage of the selection process. Main reasons for exclusion during the full-text screening stage will be documented.

### Data extraction

Using study-specific data forms, pre-determined data will be extracted for each study. Data extracted will include:

1. Study characteristics (author names and contact information, year, institution, country, language, and source of funding)
2. Study design
3. Population characteristics (e.g., sample size, age at diagnosis, degree of hearing loss, and permanent bilateral/unilateral loss)
4. Details of control or comparison groups (if available according to the study design)
5. Type of assessment
6. Exposure—risk factors (including any author-specific definitions)
7. Onset of hearing loss (e.g., proportion with congenital, early, late onset including author-specific definitions)
8. Progressive loss (including author-specific definitions)
9. Outcome data (including author-specific definitions of hearing loss)

One researcher will extract all information, which will be verified by a second reviewer. Discrepant findings will be resolved through consensus or a third reviewer when required.

### Quality assessment

The risk of bias assessment will be conducted by one researcher and verified by a second. Randomized control trials (RCT) will be evaluated using the Cochrane Risk of Bias Tool (https://methods.cochrane.org/bias/resources/cochrane-risk-bias-tool). Other study designs will be assessed using the Qualitative Assessment Tool for Quantitative Studies (Effective Public Health Practice Project, https://merst.ca/ephpp/). This tool, developed at McMaster University, provides an overall methodological rating (weak, moderate, or strong) based on an appraisal of eight domains: selection bias, study design, confounders, blinding, data collection methods, withdrawals and dropouts, intervention integrity, and analysis.

### Evidence synthesis

Study characteristics will be summarized narratively in summary tables in the report; data will be presented as frequencies and percentages, means and standard deviations, or medians and interquartile ranges, as appropriate. It is anticipated that a full meta-analysis will not be possible due to heterogeneity in research designs and variability in study and reporting methods. A narrative synthesis of the evidence will be conducted when quantitative pooling of data is not possible. We anticipate that results will be organized in tables according to individual risk factors (e.g., type or onset of risk factors, onset of hearing loss). Other classifications to be considered are the types of hearing loss (e.g., sensorineural, auditory neuropathy spectrum disorder, mixed and permanent conductive disorders) and age of onset (preschool versus school age).

The feasibility of meta-analysis will be determined based on the similarity of clinical population, same risk factors, type of outcomes, and study designs. If meta-analysis is possible for some outcomes, we will use a random effects model to aggregate results. For continuous data, we will compute mean or standardized mean differences with 95% confidence intervals (CIs). For dichotomous data, risk ratios with 95% CIs will be computed, where appropriate. Statistical heterogeneity will be evaluated using *I*^2^ statistics; for the interpretation of *I*^2^, a rough guide of low (0–25%), moderate (25–50%), substantial (50–75%), and considerable (75–100%) heterogeneity will be used [27]; potential reasons for heterogeneity will be explored. If there is considerable heterogeneity, (> 75%), we will not conduct a pooled analysis. If data permit, sensitivity analyses will be undertaken with respect to risk of bias; assessing of low risk of bias will be compared against high risk/unclear risk of bias, funnel plots will be generated to assess for publication bias if sufficient numbers of studies are available for meta-analysis [27].

### Subgroup analyses

We will examine the following variables in subgroup analyses. Subgroups will be specific to age at onset (congenital and early infancy vs later childhood) and to specific conditions or treatments such as cCMV infection, meningitis, and pediatric cancers treated with ototoxic medication.

### Quality of evidence

After data extraction and analysis, we will assess the quality and strength of the overall body of evidence using the Grading of Recommendations Assessment, Development and Evaluation (GRADE) tool (http://www.gradeworkinggroup.org) [28]. To use GRADE, a summary of findings tables will be created. We will rate the evidence for both the primary outcome (presence of bilateral or unilateral hearing loss) and for the secondary outcome of progressive hearing loss. These outcomes will be assessed across the five domains included in GRADE: risk of bias, consistency, directness, precision, and publication bias. Based on these assessments, we will rate the quality of the evidence as high (very confident that the outcome is related to the risk factor), moderate (moderately confident that outcome is related to risk factor), or low (limited confidence in the outcome/risk factor association), or very low (little confidence in the outcome/risk factor association). Consensus will be reached by the research team comprised of researchers and clinicians, and the quality of evidence will guide the strength of the recommendations.

## Discussion

The primary objective of this review is to collect and synthesize information on risk factors associated with congenital, early, or late onset permanent hearing loss in children. It is widely acknowledged that children with congenital or neonatal risk factors need to be tested during the neonatal period and be closely monitored for late onset hearing loss. Our question is of importance because most newborn hearing screening and surveillance programs are based on the 2007 JCIH factors. This list was released more than 10 years ago, and its development process was not as accurate and transparent as in a systematic review. In addition, this list is modified in several programs suggesting that it does not apply to their context or practice or it is not followed in its entirety [29–32].

We intentionally developed a search strategy and included numerous study designs to capture as many risk factors as possible. Based on other reviews, it is expected that the majority of studies will be descriptive and include small samples or cohorts of children with hearing loss. Our aim is to provide recommendations for policy makers and clinics based on the best available evidence. Consequently, limiting this review to high-quality methodological designs such as randomized controlled trials or large cohorts would limit the evidence for decision-making. Because it is intended to gather evidence for clinical practitioners and policymakers, we are convinced that all types of data are useful, on the condition that their methodological strengths and weaknesses are assessed, and integrated into the results.

The selection of publication date for inclusion in this systematic review was a balanced decision. It has to be long enough to include well-known risk factors that have not been widely investigated recently, such as congenital rubella syndrome. On the other hand, it has to integrate the latest knowledge due to the evolution of healthcare and treatments over time. For example, care for premature newborns in neonatal intensive care units has changed and practice in the administration of ototoxic drugs is better controlled, which has a positive impact on acquired hearing loss. Consequently, the systematic review aims to integrate both knowledge that has not been recently investigated and new scientific data on the possible association between risk factors and hearing loss. Only risk factors included in the review articles will be addressed, that is, risk factors not included in articles covered by our comprehensive search strategy will not be considered for hearing loss in children.

We anticipate that the results from this systematic review will guide decision-making in the Ontario IHP and will be applicable to UNHS programs worldwide.

## Additional files

Additional file 1: PRISMA-P 2015 Checklist. (DOCX 30 kb)

Additional file 2: Search strategy to be used. (PDF 330 kb)

## Data Availability

The datasets used and/or analyzed during the current study are available from the corresponding author on request.

## Abbreviations

cCMV: Congenital cytomegalovirus
EHDI: Early hearing detection and intervention
IHP: Infant Hearing Program
JCIH: Joint Committee on Infant Hearing
PRISMA-P: Preferred Reporting Items for Systematic review and Meta-Analysis Protocols
UNHS: Universal newborn hearing screening
