# Supplementary material for: Risk factors for hearing loss in children: a systematic literature review and meta-analysis protocol
Source: Syst Rev. 2019 Jul 17;8:172. doi: 10.1186/s13643-019-1073-x (PMC6637473; doi:10.1186/s13643-019-1073-x)
Supplement: Supplementary file 2 — Search strategy to be used. (PDF 330 kb) [file 13643_2019_1073_MOESM2_ESM.pdf]

Electronic search strategies

Note: Searches were conducted using an Ovid multi-database search and duplicate records were removed online giving preference to MEDLINE, then Embase, with no field preference. Lines 1-8 are optimized for MEDLINE and the main question constructs are broken out in separate lines for clarity. Lines 9-18 are optimized for Embase. The next lines isolate the records to the database the search was designed for, combine those sets and then remove duplicate records and final isolate the records from each database again so each can be downloaded and imported into the citation manager using a database-specific import filter.

1. exp Hearing Loss/ or (hearing loss or hearing impair* or deaf* or hypoacus*).ti,ab,kf.
2. exp Infant, Premature, Diseases/ or exp Infant, Premature/ or exp Hypoxia/ or exp Respiratory Distress Syndrome, Newborn/ or exp Hypertension, Pulmonary/ or exp Persistent Fetal Circulation Syndrome/ or exp Hernias, Diaphragmatic, Congenital/ or exp Hypoxia-Ischemia, Brain/ or Cerebral Intraventricular Hemorrhage/ or exp Leukomalacia, Periventricular/ or exp Extracorporeal Membrane Oxygenation/ or exp High-Frequency Ventilation/ or Nitrous Oxide/ or exp Cytomegalovirus Infections/ or exp Toxoplasmosis, Congenital/ or exp Herpes Simplex/ or exp Toxoplasmosis/ or exp Rubella/ or exp Syphilis/ or exp Meningitis/ or exp HIV Infections/ or exp Measles/ or exp Mumps/ or exp Neonatal Sepsis/ or exp Hyperbilirubinemia/ or exp Exchange Transfusion, Whole Blood/ or exp Craniofacial Abnormalities/ or Cleft Lip/ or Ear/ab or exp Cisplatin/ or exp Down Syndrome/ or Stickler.mp. or Branchio-oto-renal Syndrome/ or CHARGE Syndrome/ or Waardenburg Syndrome/ or Treacher Collins.mp. or exp Osteogenesis Imperfecta/ or exp Neurofibromatosis 2/ or Alport.mp. or Acrocephalosyndactylia/ or exp Mucopolysaccharidosis II/ or Crouzon syndrome.mp.
3. Risk/ or Risk Assessment/ or Risk Factors/ or risk.ti,ab,kf.
4. exp Cohort Studies/ or cohort$.tw. or Controlled Clinical Trial.pt. or exp Case-Control Studies/ or (case$ and control$).tw.
5. ((randomized controlled trial or controlled clinical trial).pt. or randomized.ab. or placebo.ab. or clinical trials as topic.sh. or randomly.ab. or trial.ti.) not (exp animals/ not humans.sh.)
6. (child* or adolescent* or infan*).mp. and journal article.pt.
7. 1 and (2 or 3) and (4 or 5) and 6
8. limit 7 to yr="1990 -Current"
9. exp Hearing Loss/ or (hearing loss or hearing impair* or deaf* or hypoacus*).ti,ab,kw.
10. exp Prematurity/ or exp Newborn Disease/ or Persistent Pulmonary Hypertension/ or exp Hernias, Diaphragmatic, Congenital/ or exp Hypoxia-Ischemia, Brain/ or Cerebral Intraventricular Hemorrhage/ or exp Leukomalacia, Periventricular/ or exp Extracorporeal Membrane Oxygenation/ or exp High-Frequency Ventilation/ or Nitrous Oxide/ or exp Cytomegalovirus Infections/ or exp Toxoplasmosis, Congenital/ or exp Herpes Simplex/ or exp Toxoplasmosis/ or exp Rubella/ or exp Syphilis/ or exp Meningitis/ or exp HIV Infections/ or exp Measles/ or exp Mumps/ or exp Neonatal Sepsis/ or exp Hyperbilirubinemia/ or Exchange Blood Transfusion/ or exp Craniofacial Malformation/ or exp Cleft Lip/ or exp Ear Malformation/ or Cisplatin/ or exp Down Syndrome/ or Stickler Syndrome/ or Syndrome CHARGE/ or Pendred Syndrome/ or Waardenburg Syndrome/ or Mandibulofacial Dysostosis/ or Osteogenesis Imperfecta/ or Neurofibromatosis Type 2/ or Branchiootorenal Syndrome/ or Alport Syndrome/
11. Risk/ or Risk Factor/ or risk.ti,ab,kw.
12. exp Cohort Analysis/ or exp Longitudinal Study/ or exp Prospective Study/ or exp Follow Up/ or cohort$.tw. or exp Case Control Study/ or (case$ and control$).tw.

13. (random$ or factorial$ or crossover$ or cross-over$ or placebo$ or (doubl$ adj blind$) or (singl$ adj blind$) or assign$ or allocat$ or volunteer$).mp. or crossover-procedure/ or double-blind procedure/ or randomized controlled trial/ or single-blind procedure/
14. (baby or babies or newborn* or infan* or neonat* or preschool* or pre-school* or child* or pediatr* or paediatr* or teen* or adolescen*).mp.
15. 9 and (10 or 11) and (12 or 13) and 14
16. limit 15 to conference abstract
17. 15 not 16 not exp *Hearing Aid/
18. limit 17 to (embase and yr="1990 -Current")
19. 8 use medall
20. 18 use emczd
21. 19 or 20
22. limit 21 to yr="1990 - 2000"
23. limit 21 to yr="2001- 2019"
24. remove duplicates from 23
25. remove duplicates from 22
26. 24 or 25
27. 26 use medall
28. 26 use emczd

CINAHL

S1 ((((MH "Hearing Disorders") OR (MH "Deafness+") OR (MH "Hearing Loss, Partial+")) OR (hearing loss or hearing impair* or deaf* or hypoacus*)) AND (baby or babies or newborn* or infan* or neonat* or preschool* or pre-school* or child* or pediatr* or paediatr* or teen* or adolescen*) AND risk) AND ((cohort[TIAB] OR Cohort Studies[Mesh] OR longitudinal[TIAB] OR prospective[TIAB] OR retrospective[TIAB]) OR ((MH "Case Control Studies+") or (MH "Control Group") or (MH "Matched-Pair Analysis") or (TI (case or cases) n5 TI (control or controls)) OR (AB (case or cases) n5 AB (control or controls)) OR (TI (case or cases) n3 TI (matched)) OR (AB (case or cases) n3 AB (matched)) OR TI (control group*))) Limiters - Published Date: 19900101-; Exclude MEDLINE records
